# Self-Propelled Ice on Herringbones

**DOI:** 10.1021/acsami.5c08993

**Published:** 2025-08-14

**Authors:** Jack T. Tapocik, Venkata Yashasvi Lolla, Sarah E. Propst, Saurabh Nath, Jonathan B. Boreyko

**Affiliations:** † Department of Mechanical Engineering, 1757 Virginia Tech, Blacksburg, Virginia 24061, United States; ‡ Department of Materials Science and Engineering, Virginia Tech, Blacksburg, Virginia 24061, United States; § Department of Biomedical Engineering and Mechanics, Virginia Tech, Blacksburg, Virginia 24061, United States; ∥ Department of Mechanical Engineering, Massachusetts Institute of Technology, Cambridge, Massachusetts 02139, United States

**Keywords:** Leidenfrost, superhydrophobic, phase-change, self-propulsion, ice

## Abstract

In the Leidenfrost regime, droplets or sublimating solids can ratchet across asymmetric surface structures by viscous entrainment with the underlying vapor flow. As an extension to these liquid–vapor or solid–vapor ratchets, here, we investigate the solid–liquid self-propulsion of melting ice disks. On hydrophilic herringbones, ice disks self-propel due to the unidirectional flow of viscous meltwater. This is a more viscous analog to Leidenfrost ratchets, except now a brief start-up time is needed for the underlying channels to get filled. When the herringbone is superhydrophobic using conformal nanostructures, the ice disk partially adheres to the ridge tops such that viscous entrainment cannot induce motion. Instead, after a much longer start-up time, the ice disk suddenly dislodges and slingshots across the surface by virtue of a mismatch in Laplace pressure of the meltwater on either end of the disk.

## Graphical abstract

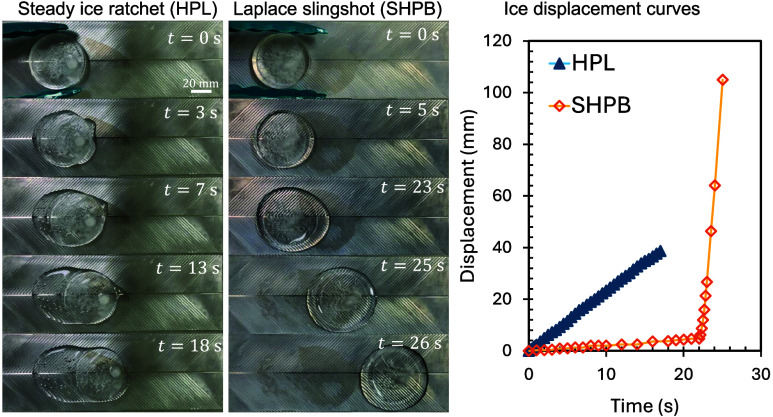

## Introduction

The Leidenfrost effect occurs when a droplet is deposited on a sufficiently superheated surface and levitates on a vapor layer generated by its own evaporation.^1–6^ An inverse Leidenfrost effect can be observed by placing a room-temperature droplet onto a cryogenic bath, where a similar vapor cushion forms beneath the droplet.^7^ Multiple studies have demonstrated that Leidenfrost droplets can self-propel across asymmetric sawtooth structures.^8,9^ Similar behavior has been observed for rigid blocks of sublimating dry ice,^10–12^ confirming that interfacial deformation is not required for propulsion. Initial debate on whether the propulsion mechanism was inertial^10^ or viscous^8^ was resolved through particle image velocimetry and scaling analysis, which confirmed the dominant role of viscous vapor flow.^13^ Leidenfrost propulsion has also been achieved using alternative geometries, such as tilted pillar arrays^14,15^ and V-shaped herringbone groove patterns,^16^ the latter being particularly advantageous due to its ease of fabrication and modeling. Wells et al. demonstrated that dry ice could power small turbines by directing the sublimation vapor flow to impinge on turbine blades.^17^ Beyond ratcheted surfaces, self-propulsion of Leidenfrost droplets has been shown on smooth substrates via Marangoni-induced circulation,^18^ on surfaces with bidirectional topographical gradients,^19^ and by breaking vapor flow symmetry through imparted initial velocities.^20^ Enhanced mobility has also been observed on dual-scale surfaces combining roughness and asymmetric structures.^21^ Notably, even irregularly shaped objects can exhibit self-propulsion under Leidenfrost conditions.^12,22^

While these Leidenfrost ratchets work for liquid–vapor or solid–vapor systems, nature has demonstrated that solid–liquid propulsion is also possible. At the Racetrack Playa in Death Valley, small (∼10 kg) boulders can mysteriously move across the desert. Early reports suggested strong gusts of wind (*U* ≈ 40 m/s) as the primary mover, with the added criteria of a wetted surface to reduce sliding friction.^23,24^ However, by the 2010s, it was recognized that boulders could move even in a moderate breeze (*U* ≈ 5 m/s).^25^ Ice rafts were therefore proposed, and in 2013, time-lapse photography confirmed that floating windowpane ice sheets, ∼10 m in diameter and 3–6 mm thick, were responsible for moving the boulders at speeds of 2–5 m/min over distances of ∼10–100 m.^26^

Jointly inspired by Leidenfrost ratchets and the Racetrack Playa, we demonstrate solid–liquid self-propulsion using ice disks on herringbones. By rectifying the flow of the underlying meltwater, we achieve self-propulsion of the solid without resorting to an external force (i.e., no wind flow). In contrast to previous reports of spontaneously rotating ice disks via asymmetric drainage from a hole,^27,28^ our self-propulsion is translational. Two distinct modes of self-propulsion were observed: ratcheting with an approximately linear displacement on hydrophilic herringbones, and a slingshot effect on nanostructured superhydrophobic herringbones due to a mismatch in Laplace pressure of the surrounding meltwater.

## Results and Discussion

Figure 1a is a visual summary of the three possible phase combinations for self-propulsion. The cases of liquid–vapor (top image) and solid–vapor (middle) Leidenfrost ratchets are already well characterized, so we focus exclusively on pioneering solid–liquid self-propulsion (bottom). Two different wettabilities were used for the enabling herringbone structure: a hydrophilic (HPL) case of bare aluminum and a superhydrophobic (SHPB) case where a nonwetting spray coating was applied (Rust-Oleum, NeverWet). A prototypical example of ice ratcheting on the HPL herringbone is shown in Figure 1b, where linear translation begins in under one second as the channels get filled with meltwater. In contrast, the ice disk on the SHPB herringbone in Figure 1c remains immobilized for ∼10 s, followed by a sudden slingshot effect (i.e., rapid acceleration). The displacement curves of these two distinct modes of self-propulsion are graphed together in Figure 1d.

**Figure 1:**
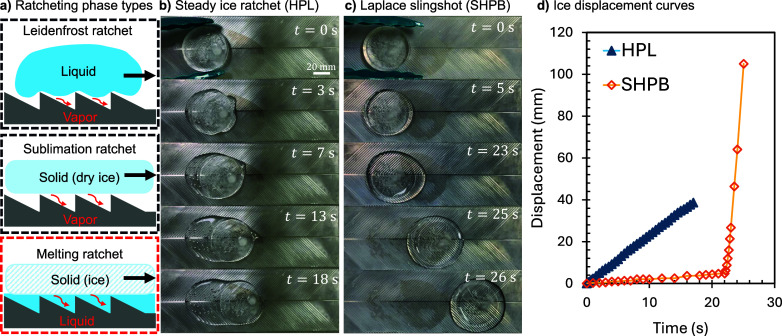
Overview of self-propelled ice disks on herringbones. (a) Conceptual schematics of the three phase combinations for ratcheting: liquid–vapor, solid–vapor, and solid–liquid. Black outlines denote previous research, while the red outline is the focus of this report. While a sawtooth pattern was drawn for visual simplicity, in actuality, a herringbone structure was used for all current experiments. (b) Top-down imaging of ice ratcheting on a HPL herringbone (surface temperature *T*_s_ = 65 °C, wedge angle α = 45°, and channel depth *h*_c_ = 0.25 mm). (c) Top-down imaging of ice suddenly slingshotting across a SHPB herringbone after a latent period of over 20 s. The system parameters were the same as in (b), aside from the change in wettability. (d) Displacement of the ice disk over time for the examples shown in (b, c).

As seen in Figure 1b, small air bubbles were observed beneath the rear portion of the ice disk and in the trailing meltwater for the hydrophilic ratchet. Prior to deposition, the ice disks contained fewer visible air bubbles. The appearance of extra bubbles within the meltwater may be due to small air pockets getting trapped within the asymmetric microgrooves during initial melting. In contrast, extra bubbles were not observed for the SHPB case, where the Cassie wetting state in both the grooves and the conformal nanostructure allows escape pathways for the air. Also exclusive to the hydrophilic substrate was the occasional rotation of the ice disk, especially near the end of a trial as the ice mass gets depleted and the ratcheting speed slows down. This may be analogous to recent reports observing gyroscopic rotation for boiling droplets on microstructured surfaces^29^ or for Leidenfrost droplets on heterogeneous wettability patterns.^30^

To more fully characterize this wettability-dependent self-propulsion, multiple system parameters were varied as shown in Figure 2. Four different herringbone geometries were used, where the wedge angle (α = 22.5° or 45°) and channel depth (*h*_c_ = 0.25 mm or 0.5 mm) were systematically varied. Two different substrate temperatures were chosen, *T*_s_ = 65 °C or 150 °C to see how the melting rate influenced the ice kinematics. The initial ice disk radius (*R*_ice,i_ = 26 mm) and height (*h*_ice_ = 10 mm), as well as the width of the herringbone channels (*w*_c_ = 1.5 mm) and intermediate ridges (*w*_r_ = 0.5 mm) were fixed across all experiments on the HPL herringbones. The active (i.e., under-ice) length of each channel is defined as *l*_*i*_, where the *i* index considers each separate channel that corresponds directly to the wedge angle and ice disk radius. Exclusively for the SHPB herringbones we tested two additional disk sizes: smaller disks where *R*_ice,i_ = 17.5 mm and *h*_ice_ = 8 mm, and larger disks where *R*_ice,i_ = 31 mm and *h*_ice_ = 10 mm.

**Figure 2:**
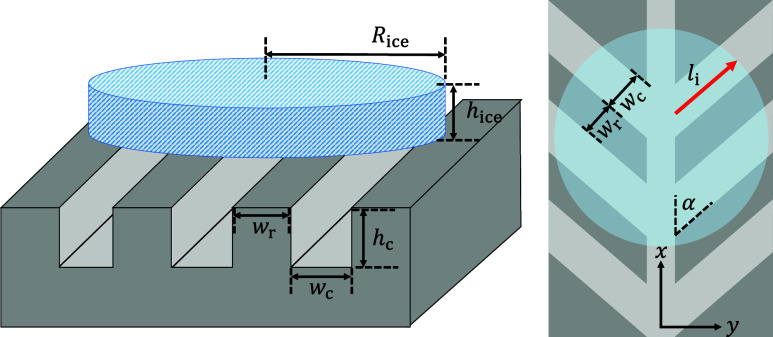
Geometric parameters for the ice disk and aluminum substrates. Left image: isometric schematic defining the channel width (*w*_c_) and height (*h*_c_), ridge width (*w*_r_), and initial ice disk height (*h*_ice_) and radius (*R*_ice,i_). Right image: top-view schematic of the ice disk (semitranslucent blue circle) over the herringbone structure (not to scale). The length of an active channel (*l*_*i*_) is denoted by the red arrow.

The six sets of conditions where self-propulsion was successful are presented in Table 1. Three successful trials were performed for each case and all experimental values given here represent an average across successful trials with error bars correlating to the standard deviation. For Cases 3 and 6, propulsion occurred for the HPL case but not the SHPB one due to the increased channel depth. Time-lapse photography and graphs of the ice disk’s displacement versus time can be seen for all six 52 mm ice disk cases in Figures S1–S6 and Movies S1 and S2. Given the profound differences in ice-meltwater interactions on the HPL versus SHPB herringbones, we will now treat each in turn.

**Table 1:** Cases of Successful Self-Propulsion

Case #	Wettability	*T*_s_ (°C)	α	*h*_c_ (mm)
1	HPL and SHPB	65	22.5°	0.25
2	HPL and SHPB	65	45°	0.25
3	HPL	65	22.5°	0.5
4	HPL and SHPB	150	22.5°	0.25
5	HPL and SHPB	150	45°	0.25
6	HPL	150	22.5°	0.5

### Hydrophilic Model

For the HPL herringbone, the channel filling time was *t*_fill_ ∼ 0.1 s for the surface temperatures used here. The subsequent translation of the ice disk was by viscous entrainment, analogous to Leidenfrost ratchets but with a liquid flow rather than a vapor one. The entrainment force was measured by integrating the inertia of the ice disk over the time it was accelerating. The characteristic magnitude of the viscous entrainment force was *F*_*μ*,e_ ∼ *F*_*i*_ ∼ 1 mN. The magnitude of *F*_*μ*,e_ increased from *T*_s_ = 65 °C to 150 °C, for the same trial parameters. For experimental rigor, trials of *T*_s_ = 220 °C were also performed but ultimately were discarded for experimental uncertainty which we attribute to nucleate boiling disrupting the flow. (Previous reports have shown that nucleate boiling is largely suppressed in the meltwater beneath ice for *T*_s_ ≤ 150 °C.)^31,32^ This acceleration time-scale was only of order *t*_accel_ ∼ 0.01 s, as the inertia was quickly consumed by what we hypothesize is viscous drag (*F*_*μ*,d_) in the thin film between the ridges and ice disk. A transition regime, from pure inertia to terminal velocity, typically lasted for *t*_trans_ ∼ 1 s. Finally, the ice disk moved with an (approximately) terminal velocity for an additional *t*_term_ ∼ 1–10 s, after which, the ratcheting ceased due to the ice disk becoming too small. Terminal velocities were of order *U*_ice_ ∼ 1 mm/s, with the exact magnitude increasing with *T*_s_ and, for a given temperature, being higher for α = 45° than 22.5°. By comparison, a Leidenfrost droplet on a herringbone ratchets at *U*_drop_ ≈ 50–150 mm/s,^16^ up to two orders of magnitude faster, which we attribute to the dramatic decrease in density for water vapor and higher substrate temperature used for the Leidenfrost regime.

An analytical model for the HPL ice ratchet balances thermal conduction and latent heat: *k*_w_ *w*_r_ *l*_*i*_Δ*T*/*h*_e_ = *ṁ* *L*_f_, where *k*_w_ is the thermal conductivity of the meltwater, *w*_r_ *l*_*i*_ is the cross-sectional area of a ridge top, Δ*T* = *T*_s_ – *T*_ice_ where *T*_ice_ ≈ 0 °C, *h*_e_ is the thickness of the thin excess meltwater film between the ridge tops and the bottom of the ice disk where conduction is dominant, *ṁ* is the mass flow rate of melting above a given ridge top, and *L*_f_ ≈ 334 kJ/kg is the latent heat of melting. We can neglect thermal conduction from the channels: *q*_ridge_/*q*_chan_ ≈ (*h*_c_ + *h*_e_)/*h*_e_ ≈ 8 for *h*_c_ = 0.25 mm and *q*_ridge_/*q*_chan_ ≈ 15 for *h*_c_ = 0.5 mm, respectively. We assume Poiseuille flow between two parallel plates, i.e., each ridge top and the overlying ice (Figure 3a). The pressure gradient driving meltwater into the channels is –*dP*/*dx ≈ ρ*_i_g*h*_ice_/(*w*_r_/2), where ρ_i_ is the density of ice and $w_{r}/2$ is the symmetric half-space of a ridge. Collectively, this results in an estimation of the thickness of the excess meltwater:

$$h_{e}={\left(\frac{3\mu_{w}w_{r}^{2}k_{w}\Delta T}{\rho_{w}L_{f}\rho_{i}gh_{\mathrm{ice}}}\right)}^{1/4}$$ 1

where μ_w_ and ρ_w_ are the viscosity and density of the meltwater. Calculated values range from *h*_e_ ≈ 35–48 μm for the parameter space used. While *h*_e_ could not be experimentally measured here, this is consistent with *h*_e_ ∼ 10 μm deduced from a separate work by measuring the heat flux of meltwater beneath ice.^32^

**Figure 3:**
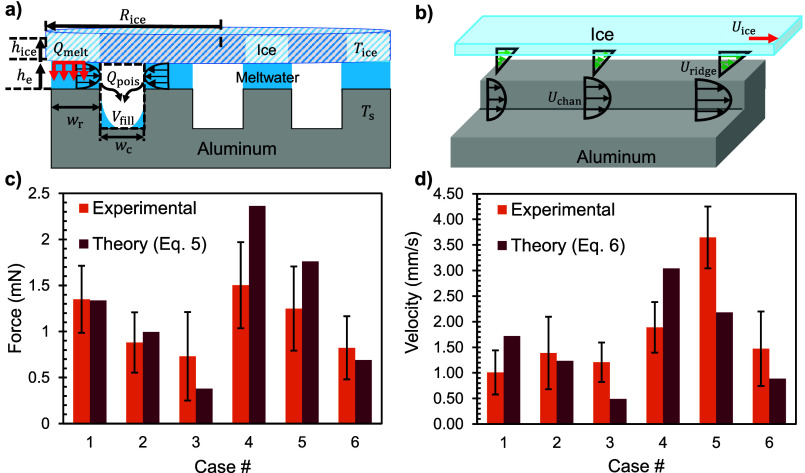
Modeling and results for the HPL ice ratchet. (a) Side-view schematic of initially filling the channels with meltwater. Thermal conduction across the ridge tops facilitates the volumetric flow rate of melting that then drains into the channels via a planar Poiseuille flow (i.e., *Q*_melt_ ≈ *Q*_pois_). (b) Side-view depiction of the viscous resisting flow along the ridge tops during steady-state ratcheting at speed *U*_ice_. Both the channel velocity, *U*_chan_, and the viscous resisting flow, *U*_ridge_, are increasing along the active channel lengths. (c) Comparison of the ice disk’s initial ratcheting inertia for experiments (orange bars) and theory (burgundy, using eq 5). (d) Bar graph depicting the terminal velocity as measured experimentally (orange) and calculated theoretically with eq 6 (burgundy). All error bars are calculated using standard deviation across the three working trials.

The filling time for ratcheting onset can be estimated by

$$t_{\mathrm{fill}}=\frac{\rho_{w}V_{\mathrm{fill}}}{ṁ}=\frac{3\mu w_{r}w_{c}H}{h_{e}^{3}\rho_{i}gh_{\mathrm{ice}}}$$ 2

where *V*_fill_ = *w*_c_ *l_i_* *H* is the required overfilling volume of a channel, *H* = *h*_c_ + *h*_e_ accounts for the overlying excess, and eq 1 is used for expressing *h*_e_. For the case of *h*_c_ = 0.25 mm, eq 2 results in *t*_fill,t_ ≈ 0.28 s and 0.15 s for *T*_s_ = 65 °C and 150 °C, respectively. This is in excellent agreement with the respective experimental values of *t*_fill,e_ ≈ 0.23 s and 0.13 s for α = 45°. See Figure S7 for a graphical comparison of all *t*_fill_ values.

To solve for the viscous entrainment force, the velocity of the meltwater needs to be solved as a function of *x*_*i*_, where the *x*-axis now lies along each herringbone channel and *i* is still the index of all active channels beneath the ice disk. Accounting for meltwater entering into the channel from the ridge tops, mass conservation results in $[U_{\mathrm{chan}}Hw_{c}{]}_{x+\mathrm{dx}}-[U_{\mathrm{chan}}Hw_{c}{]}_{x}=2U_{\mathrm{pois}}h_{e}dx$ , where *U*_chan_ is the x-dependent axial channel velocity averaged across *Hw*_c_ and *U*_pois_ is the averaged velocity of meltwater as it spills over either ridge top. Defining *x*_*i*_ = 0 at the start of each channel (i.e., beginning of either branch) and taking a first-order Taylor series expansion:

3 $$U_{\mathrm{chan}}=\frac{2k_{w}\Delta T}{\rho_{w}L_{f}Hw_{c}}x$$

Assuming a parabolic flow profile across *H* (Figure 3b), where *U*_max_ = 1.5*U*_chan_, the shear stress at the ice disk is given by $\mu_{w}\frac{\partial u}{\partial z}{|}_{z=H}=6U_{\mathrm{chan}}/H$ . The Reynolds number for flow within a rectangular channel is given by *Re* = ρ_w_ *U*_chan_ *D*_h_/μ_w_, where *U*_chan_ is the characteristic Poiseuille velocity, and *D*_h_ = 2*w*_c_ *h*_c_/(*w*_c_ + *h*_c_) is the hydraulic diameter of the channel. Depending on the channel height and the temperature, Re ≈ 3–14 where the smaller channel and lower temperature correspond to the lowest Re. In conjunction with eq 3, we arrive at the entrainment force for a single channel of covered length *l*_i_:

4 $$F_{\mathrm{chan},i}=\frac{6\mu k_{w}w_{r}\Delta Tl_{i}^{2}}{\rho_{w}L_{f}h_{e}H^{2}}$$

The total entrainment force is now the sum of all active channels underneath the ice disk:

$$F_{\mathrm{entrain}}=\sum_{i=1}^{n}\frac{6\beta \mu k_{w}w_{r}\Delta Tl_{i}^{2}}{\rho_{w}L_{f}h_{e}H^{2}}\cos\alpha$$ 5

where cos α is the component in the direction of ice displacement (*x*-axis in Figure 2) and *β* ≈ 0.25 is a semiempirical fitting factor accounting for losses including potential decentering of the ice disk during motion, partial adhesion of the ice disk to the herringbone, nonuniformities in the excess meltwater thickness, and bubbles beneath the disk. Naively assuming that the beginning of a herringbone channel is perfectly aligned with the back edge of the ice disk, we solve geometrically for the length of every channel *l_i_* beneath the ice (see Figure S8). For *h*_c_ = 0.25 mm and α = 45°, eq 5 results in *F*_entrain,t_ ≈ 0.99 mN and 1.8 mN for *T*_s_ = 65 °C and 150 °C, respectively. This agrees reasonably well with the respective experimental values of *F*_entrain,e_ ≈ 0.89 mN and 1.3 mN obtained by measuring the initial inertia (Figure 3c).

After the inertia has dissipated, the terminal velocity of the ice disk (*U*_ice_) is obtained by balancing *F*_chan,*i*_ = *F*_ridge,*i*_, where the latter is the resisting shear force in the excess meltwater above the ridge tops. We assume a linear velocity profile of the viscous resisting flow, *U*_ice_/*h*_e,dyn_, where *h*_e,dyn_ is the (reduced) thickness of the excess meltwater layer during dynamic ice movement. Balancing the two viscous forces by considering eq 4 and solving for the terminal ratcheting speed:

6 $$U_{\mathrm{ice}}=\frac{6k_{w}\Delta T{\mathrm{l̅}}_{i}}{\rho_{w}L_{f}H^{2}}h^{*}$$

where h* = *h*_e,dyn_/*h*_e_ is the nondimensional height of the dynamic excess meltwater. We average *l_i_* so that a characteristic length of an active channel is used to solve for the ice disk velocity. For example, for our size ice disk, the wedge angle of α = 22.5° results in a average under-ice channel length of l̅i ≈ 29.7 mm, compared to l̅i ≈ 23.9 mm for α = 45° (see Figure S8). We used a fixed *h*_e,dyn_ ≈ 0.28 μm as a semiempirical value that best fits *U*_ice,t_ to *U*_ice,e_ across all experimental parameters. The terminal velocity decreases with wedge angle and increases with temperature. Again considering *h*_c_ = 0.25 mm and α = 45°, we calculated initial velocities of *U*_ice,t_ ≈ 1.2 mm/s and 2.2 mm/s for *T*_s_ = 65 °C and 150 °C, respectively. These values are extremely close to the experimental velocities of *U*_ice,e_ ≈ 1.4 mm/s and 1.9 mm/s (Figure 3d).

Propulsion of the ice disk requires its initial thickness to be sufficient to both fill the underlying channels with meltwater and provide enough hydrostatic pressure to drive the flow along the channels. The void fraction, *ε*, is the top-down projected fraction of the surface comprised of open channels: *ε* ≈ *w*_c_/(*w*_c_ + *w*_r_) ≈ 0.75. Solving for the minimal height of the ice to fill the channels, we arrive at *h*_ice_ ≈ ρ_w_ *εh*_c_/ρ_i_ ≈ 0.21 mm for the case of *h*_c_ ≈ 0.25 mm. Some additional ice thickness would be required to subsequently drive the meltwater along the channels.

It is instructive to compare the dynamics of our solid–liquid viscous ratchet to the established case of a solid–vapor Leidenfrost ratchet (i.e., sublimating dry ice).^10^ The characteristic force and velocity for our ice disk configuration (*R*_ice_ ≈ 26 mm, *h*_ice_ ≈ 10 mm) are on the order of *F*_entrain_ ∼ 1 mN and *U*_ice_ ∼ 1 mm/s, respectively. In contrast, dry ice ratcheting across a sawtooth structure resulted in characteristic values of *F*_entrain_ ∼ 0.1 mN and $U_{{\mathrm{CO}}_{2}}$ ∼ 100 mm/s for a comparable height of $h_{{\mathrm{CO}}_{2}}\approx 10\,$ mm but with a smaller radius, $R_{{\mathrm{CO}}_{2}}\approx 6.5$ mm.^13^ Thus, our system exhibits an order-of-magnitude increase in the driving force but a two-order-of-magnitude decrease in velocity. These differences are primarily due to the much higher viscosity (μ_w_ ≈ 1 mPa·s) and density (ρ_w_ ≈ 1000 kg/m^3^) of liquid water relative to that of CO_2_ vapor ( $\mu_{{\mathrm{CO}}_{2}}\approx$ 0.03 mPa·s and $\;\rho_{{\mathrm{CO}}_{2}}\approx$ 1 kg/m^3^), as well as differences in the substrate temperature and boiling point of the solid.

### Superhydrophobic Model

For the SHPB herringbone, two dramatic changes were observed. First, the onset time of *t*_onset_ ∼ 10 s for propulsion to initiate was two orders of magnitude longer than the HPL case. Second, the sudden burst of propulsion was dramatic and more slingshot-like. Figure 4 includes time-lapse photography and graphs of the ice disk’s displacement versus time for the three different radii on the SHPB surface. The slingshot effect was qualitatively similar in all cases, but with shorter onset times and faster accelerations for smaller ice disks. The much longer onset time, and size-dependent slingshot acceleration, will each be rationalized with analytical models.

We hypothesize that meltwater filling the herringbone channels is no longer sufficient for entrainment because the SHPB coating tends to displace the excess meltwater, sticking the ice disk to portions of the ridge tops. Neither is buoyancy sufficient to overcome the ice adhesion, as ice disks always remained immobilized even after getting completely submerged in a surrounding ring of meltwater. Rather, takeoff could only occur after viscous entrainment, buoyancy, and a completely new driving force worked in tandem to dislodge the ice from the ridge tops. Once detached, it is clear from the displacement curves in Figure 1d that this new, slingshot-like driving force is dominant in magnitude over viscous entrainment. Visually, it is evident that the new driving force is a mismatch in Laplace pressure between a flat puddle extending from the leading edge of the ice disk versus a curved ring at the trailing edge.

**Figure 4:**
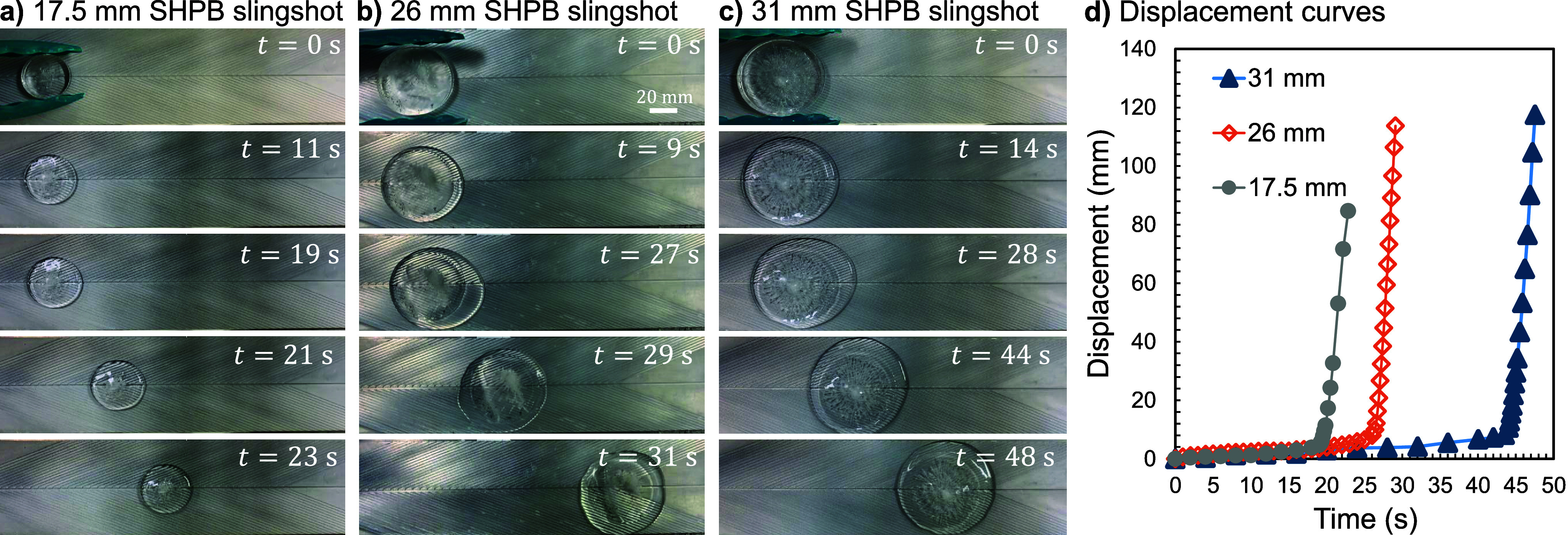
Representative trials of SHPB Case 1, with surface temperature *T*_s_ = 65 °C, herringbone angle α = 22.5°, and channel depth *h*_c_ = 0.25 mm. (a–c) Time-lapse photography of a trial with initial radius (a) *R*_ice,i_ = 17.5 mm, (b) 26 mm, or (c) 31 mm. (d) Graph of displacement versus time with the corresponding *R*_ice,i_ = 17.5 mm trial in gray circles, *R*_ice,i_ = 26 mm trial trial in hollow orange diamonds, and *R*_ice,i_ = 31 mm in blue triangles.

Therefore, we model the onset time as the melting time required to generate the leading puddle. This can be broken down into three separate meltwater volumes, as shown in Figure 5a. The first required volume to fill is the underlying channels. This is analogous to *t*_fill_ from the HPL case, with the difference of the meltwater being in the Cassie state with respect to the conformal nanoroughness. Regardless, the meltwater is still almost entirely filling the volume of the channels themselves: *V*_1_ ≈ Σ_*i*_ *l*_*i*_ *Hw*_c_. The second volume is a ring of water about the ice disk, whose lateral extent scales with the capillary length (*l*_c_): *V*_2_ ≈ π­(2*R*_ice,i_ *l*_c_ + *l*_c_^2^)*h*_ice_. This ring forms because, after emerging from the underlying channels, the meltwater prefers adhering to the sides of the hydrophilic ice disk to minimize wetting the SHPB herringbone. The last volume is the preferential growth of a puddle in front of the disk extending beyond the ring, where the puddle asymmetry is due to the herringbone grooves not allowing for backflow. Due to the high apparent contact angle of meltwater on the SHPB surface, *θ* ≈ 160°, this volume can be approximated as an oval cross-section extruded about a partial arc length of the ice disk. Analytically, this comes out to *V*_3_ ≈ (3/2)­π*l*_c_^2^ *R*_ice,i_ θ, where *θ* ≈ 2π/3 is the arc length of the puddle (see Supporting Information).

**Figure 5:**
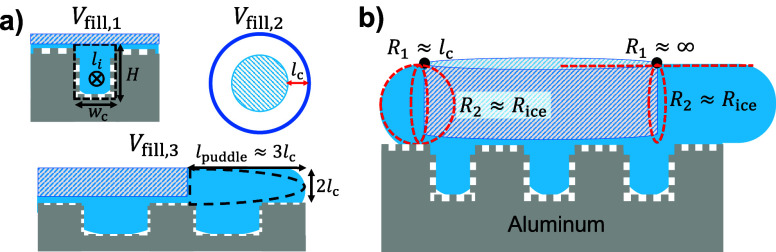
Modeling for the SHPB ice slingshot. (a) Three meltwater filling volumes required to enable the Laplace slingshot. White spaces underneath the meltwater represent air pockets entrapped within the conformal nanotexture. *V*_fill,1_ is the channel filling time (where the nanopillars do not affect the volume significantly), *V*_fill,2_ is the volume to complete a ring around the periphery of the ice disk, and *V*_fill,3_ is the volume to create a flat puddle preferentially in front of the ice disk. (b) Schematic of the Laplace pressure difference on the trailing (left) and leading (right) ends of the ice disk due to the preferential puddle.

Using the same balance of thermal conduction, latent heat of melting, and Poiseuille flow to solve for *ṁ*, the onset time for the Laplace force is given by

7 $$t_{\mathrm{onset}}=\frac{6\mu w_{r}}{h_{e}^{3}\rho_{i}gh_{\mathrm{ice}}l_{i}}\left(\frac{V_{1}}{n_{1}}+\frac{V_{2}}{n_{2}}+\frac{V_{3}}{n_{3}}\right)$$

where *n*_1_, *n*_2_, and *n*_3_ represent the total number of channels actively contributing to *V*_1_, *V*_2_, and *V*_3_, respectively. All channels beneath the ice disk contribute to *V*_1_ and *V*_2_ such that *n*_1_ = *n*_2_ where the wedge angles control the number of channels beneath the ice disk. To calculate *n*_3_, the active channels contributing to the puddle in front were counted between the angles θ = π/6 and 5π/6. For *h*_c_ = 0.25 mm and *T*_s_ = 150°C, eq 7 results in *t*_fill,t_ = 4.94 s for α = 22.5° and 4.92 s for α = 45°, which are in excellent agreement with the experimental time scales of *t*_fill,e_ = 5.61 s and 4.83 s where the puddle can be visibly seen to “tug” on the ice disk just prior to dislodging and takeoff. See Figure S9 for all comparisons of *t*_onset_ for each of the ice disk radii.

The slingshot force is captured by estimating the local curvature at either end of the ice disk after *t*_fill_ to find the pressure difference, as visualized in Figure 5b. The Laplace pressure at the trailing edge is *P*_L,trail_ ≈ *γ*(1/l_c_ + 1/*R*_ice,s_), where γ is the surface tension of water and *R*_ice,s_ is the somewhat reduced ice disk radius upon takeoff. Conversely, the flat puddle top extending from the leading edge results in a decreased Laplace pressure of *P*_L,lead_ ≈ γ/*R*_ice,s_. The net difference in Laplace pressure across the ice disk is then Δ*P*_L_ ≈ γ/*l*_c_. Extending this driving pressure along the area of ice-puddle contact, (2*l*_c_)­(2*πR*_ice,s_/3), yields the capillary driving force:

8 $$F_{\mathrm{cap}}\approx \frac{4\pi }{3}\gamma R_{\mathrm{ice},s}$$

The resulting slingshot inertia is the balance of *F*_i_ ∼ *F*_cap_ and is independent of the wedge angle. Extending the force into an acceleration, we divide by the mass of the ice disk at takeoff.

9 $$a_{\mathrm{disk}}\approx f\frac{4\gamma }{3\rho_{i}R_{\mathrm{ice},s}h_{\mathrm{disk}}}$$

where *f* ≈ 0.15 is an empirical fitting factor that is likely much less than unity due to losses from ice adhesion, ice sliding friction, or viscous stress in the excess film.

Figure 6 graphs the initial slingshot acceleration as a function of *R*_ice,s_, where eq 9 is in excellent agreement with experimental measurements. The model uses a fixed value of *h*_disk_ ≈ 5 ± 0.5 mm as the characteristic ice disk height at takeoff, as estimated from side-view photography. Recall that three different ice disk radii were used experimentally; top-down imaging revealed that the *a*_disk_ = 17.5, 26, and 31 mm disks melted down to *R*_ice,s_ ≈ 17, 25, and 30 mm when the Laplace slingshot initiated. These experiments confirm the nonlinear trend of *a*_disk_ ∝ 1/*R*_ice,s_ predicted by eq 9. The experiments also validate that *a*_disk_ is independent of the melt rate or wedge angle, as evidenced by a near-constant value of *a*_disk,e_ = 61 ± 7 mm/s^2^ for the *R*_ice,s_ ≈ 25 mm disk averaged across Cases 1, 2, 4, and 5 (uncertainty corresponding to a standard deviation). This agrees with the substrate-independent prediction of *a*_disk,t_ = 63 ± 7 mm/s^2^ from eq 9. After takeoff, there is a decrease in both the Laplace force, due to the redistribution of the leading puddle, and the effective sliding friction, due to the successful dislodging of the ice disk. This results in a quasi-terminal velocity of *U*_SHPB_ ≈ 20–60 mm/s, approaching that of a Leidenfrost droplet ratchet.

**Figure 6:**
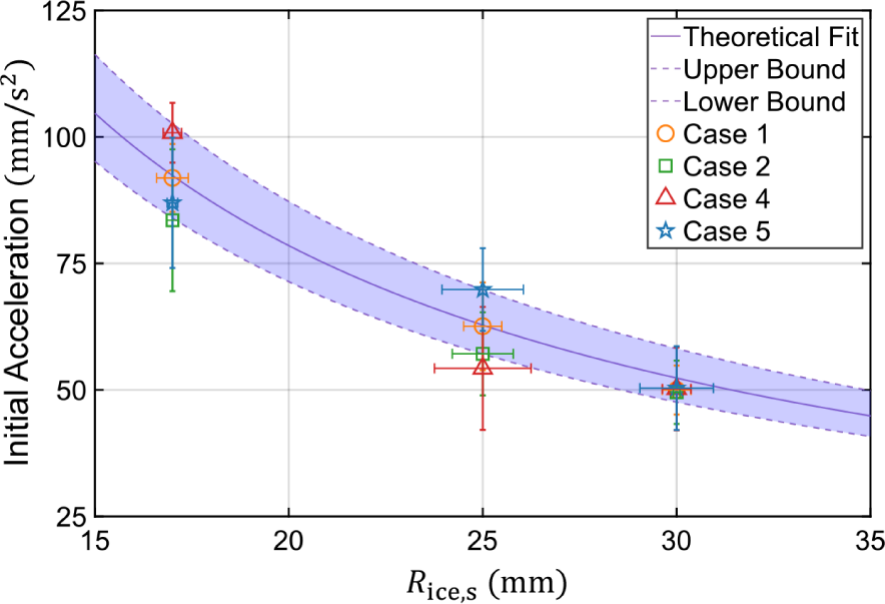
Graph comparing the theoretical (eq 9) and experimental values for the initial accleration of slingshotting ice disks on the superhydrophobic herringbone.

## Control Case: Flat Plate

A set of control experiments was conducted on uniform HPL and SHPB aluminum plates without herringbones. This was to confirm that the ratcheting and slingshot mechanisms originated purely from the geometry of the herringbone structure. Five trials were performed at a substrate temperature of *T*_s_ = 65 °C, with top-down video recordings used to track the motion of the ice disk. As shown in Figure 7a, each ice disk on the HPL surface exhibited random, undirected motion, confirming that in the absence of geometric meltwater confinement, there is no directed propulsion. The velocity of the undirected motion was of order *U*_ice_ ∼ 1 mm/s, comparable to disks ratcheting on the herringbone. For the SHPB control, the ice disks were firmly adhered to the plate, such that even undirected motion could not occur (Figure 7b). This behavior confirms the important role of ice adhesion for the Laplace slingshot on the SHPB herringbone, where a considerable difference in Laplace pressure was required to build up before slingshotting could initiate. On the flat plate, the melting occurs uniformly, precluding any such Laplace mechanism for dislodging the adhered ice. A visual comparison of the random motion on HPL versus the immobility on SHPB can be seen in Movie S3.

**Figure 7:**
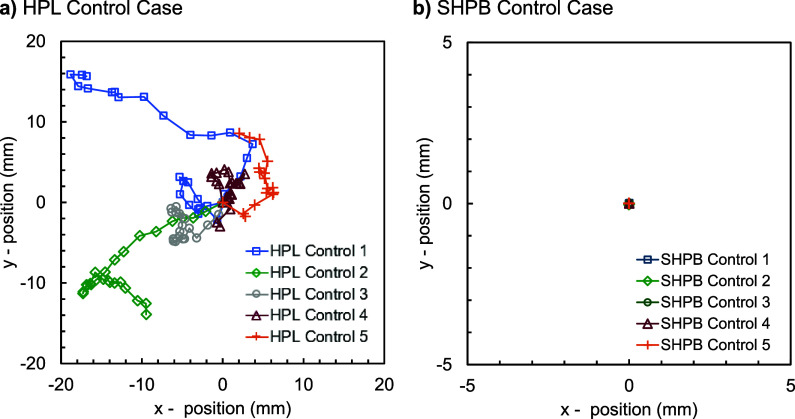
Displacement of melting ice disks on the control case of uniform plates (i.e., no herringbones) heated to *T*_s_ = 65 °C. (a) Disk motion was slow and random on a smooth HPL plate. (b) Due to ice adhesion, there was no motion of the melting ice disk on a plate with a uniform SHPB nanostructure. Trials 1–5 are represented by blue squares, green diamonds, gray circles, burgundy triangles, and orange plus signs, respectively. Neighboring data points represent an elapsed time of 1 s.

## Conclusions

We have shown that melting solids can self-propel on a herringbone structure, analogous to Leidenfrost ratchets but with surprising mutations. On a HPL herringbone, propulsion is still driven by viscous entrainment, but the inertia is dissipated by viscous drag in the excess film rather than by the soft shocks of the bumps.^16^ The resulting terminal velocity is reduced by one to two orders of magnitude, due to the much higher density of meltwater compared to water vapor. On a nanostructured SHPB herringbone, ice disks can no longer propel from viscous entrainment due to partial adhesion to the nonwetting ridge tops. After enough time has passed for the meltwater to generate a puddle in front of the ice disk, the resulting difference in Laplace pressure suddenly slingshots the ice across the surface at a higher speed comparable to Leidenfrost ratchets. Due to the collective effects of adhesion, wettability, and the increase in density and viscosity of the lighter phase, a solid–liquid ratchet therefore exhibits nontrivial and multimodal differences in propulsion compared to liquid–vapor or solid–vapor systems.

These findings demonstrate the potential for passive ice removal and phase-engineered microtransport by harnessing controlled melting and surface-guided motion, with implications for anti-icing systems, self-cleaning surfaces, and power-free microfluidic transport. Future research should be conducted to enhance the fundamental understanding of both the viscous ice ratchet and the slingshotting Laplace disk by systematically exploring solid–liquid propulsion with alternative phase-change materials (e.g., paraffin wax), more widely varied surface structure geometries, using nonuniform shapes (e.g., rectangles or nonlevel disks), and expanding the temperature range to include the three-phase Leidenfrost effect.^31^ Additionally, introducing nonlinear surface geometries may enable curved trajectories, as the ice disk naturally follows the centerline of the herringbone.

## Experimental Setup

The ice disks were fabricated by pouring distilled water in polycarbonate Petri dishes. The inner diameters of the Petri dishes were either 35 mm or 52 mm. A larger 62 mm Petri dish was 3D printed using a high temperature resin (Formlabs, Tough 1500 Resin V2). Water-filled dishes were placed in a standard freezer (*T*_∞_ ≈ −10 °C). Bottom-up freezing of the water was promoted by thermally insulating the Petri dishes, in order to block convective cooling at the upper interface while ensuring heterogeneous ice nucleation at the bottom liquid–solid interface. The benefit of bottom-up freezing is that it minimizes air bubbles while also keeping the bottom of the ice disk smooth.

The herringbone grooves were milled into aluminum plates (6061-T6). These herringbones were left uncoated for the viscous ice ratchets or coated with a commercial spray (Rust-Oleum, NeverWet) for the superhydrophobic Laplace slingshots. The SHPB coating included both a base coat and a top coat and was applied according to the manufacturer’s recommendations. Using the swell-shrink method on a goniometer (ramé-hart, Model 590), the apparent advancing and receding contact angles were measured as θ_A_ ≈ 152° and θ_R_ ≈ 151° for the SHPB aluminum and θ_A_ ≈ 77° and θ_R_ ≈ 57° for the uncoated aluminum. The ultrasmall contact angle hysteresis for the latter SHPB surfaces indicates the air-trapping Cassie state. Two different plates were used for the control case experiments each with an area of 125 × 150 mm. A thin flat cut was performed to create a level and scratch-free surface. One of the plates used the same commercial spray to make the surface SHPB.

A hot plate (Valad Electric Heating, HP24 × 36) was set to the desired temperature. Thermal paste was spread over the back face of a herringbone plate to bond it to the hot plate. A thermocouple was used to determine the slightly cooler steady-state temperature of the herringbone (*T*_s_). The herringbone plate was carefully leveled to ensure there was no angle of inclination. Immediately before use, a Petri dish was removed from the freezer and the ice disk was gently popped out. The flat bottom face of the ice disk was gently deposited onto the herringbone using rubber tongs. Simultaneous top-down imaging (iPhone XR) and side-view imaging (iPhone 6s) was captured for each ice propulsion event. This allowed for both the displacement (top-down) and height (side-view) of the ice disk to be measured over time. Image analysis was performed using an open-source software (Tracker).

## Supplementary Material









## Notes

Derivations for all equations representative trials of cases 1–6 (Figures S1–S6); comparison of the experimental onset time and theoretical filling time for ice ratcheting initiation on the HPL herringbone (Figure S7); graphical image of the active channel length (Figure S8); comparison of the experimental and theoretical filling times for ice slingshotting on the SHPB herringbone (Figure S9); top view of clockwise rotation of an ice disk on the HPL herringbone with surface temperature *T*_s_ = 65 °C (Figure S10) (PDF)
Top-view videos of the six successful cases of ice ratcheting on the HPL herringbones (MP4)
Top-view videos of the four successful cases (Cases 1, 2, 4, and 5) of ice slingshotting on the SHPB herringbones (MP4)
Top-view videos for the control experiments on uniform HPL or SHPB plates without herringbones (MP4)
