# Supplementary material for: Self-Propelled Ice on Herringbones
Source: ACS Appl Mater Interfaces. 2025 Aug 14;17(34):48873–80. doi: 10.1021/acsami.5c08993 (PMC12400281; doi:10.1021/acsami.5c08993)
Supplement: Supplementary file 1 [file am5c08993_si_001.pdf]

Supporting Information:

Self-Propelled Ice on Herringbones

Jack T. Tapocik,[†] Venkata Yashasvi Lolla,[†] Sarah E. Propst,[‡] Saurabh Nath,[¶,§] and Jonathan B. Boreyko[*,†]

†*Department of Mechanical Engineering, Virginia Tech, Blacksburg, Virginia 24061, USA.*

‡*Department of Materials Science and Engineering, Virginia Tech, Blacksburg, Virginia 24061, USA*

¶*Department of Biomedical Engineering and Mechanics, Virginia Tech, Blacksburg, Virginia 24061, USA*

§*Department of Mechanical Engineering, Massachusetts Institute of Technology, Cambridge, MA, 02139, USA*

E-mail: boreyko@vt.edu

Complete derivation of model for viscous ice ratchet

All variables are identical to those defined in the main manuscript. Assumptions for modeling ice-meltwater self-propulsion by viscous entrainment:

- Thermal conduction from the ridge tops is dominant relative to the channels and is only in z-direction, such that $q''_{\mathrm{cond}} \approx q''_{\mathrm{melt}}$ where q''_{cond} is the heat flux over the ridges.
- The radius of the ice disk, R_{ice}, is approximately constant during the initial stages of melting being modeled.
- The meltwater spills into the channels and flows unidirectionally: $Q_{\mathrm{x+dx}} - Q_{\mathrm{x}} = Q_{\mathrm{pois,dx}}$, where the right-hand side represents meltwater spilling into the channel from the overlying ridges to effectively increase Q along the length of each channel.

A first order Taylor series expansion of $Q_{\mathrm{x+dx}} - Q_{\mathrm{x}} = Q_{\mathrm{pois}}$ is solved

$$[U_{\mathrm{chan}} H w_{\mathrm{c}}]_{\mathrm{x+dx}} - [U_{\mathrm{chan}} H w_{\mathrm{c}}]_{\mathrm{x}} = U_{\mathrm{pois}} h_{\mathrm{e}} dx \tag{S1}$$

Which yields

$$dU_{\mathrm{chan}} = \frac{U_{\mathrm{pois}} h_{\mathrm{e}}}{H w_{\mathrm{c}}} dx \tag{S2}$$

Solving now for U_{chan}

$$U_{\mathrm{chan}} = \frac{2 k_{\mathrm{w}} \Delta T}{\rho_{\mathrm{w}} L_{\mathrm{f}} H w_{\mathrm{c}}} x \tag{S3}$$

Eq. S3 is the same as Eq. 3 in the main text.

To find the force of the system the explicit velocity at the ice-meltwater interface is needed.

$$u(z) = az^2 + bz + c, \text{ BCs} : u(z = 0) = 0, u(z = H/2) = U_{\mathrm{max}} \tag{S4}$$

Where U_{max} is the maximum velocity in parallelpiped channel, $U_{\text{max}} = (3/2)U_{\text{chan}}$, resulting in:

$$u(x,z) = \frac{-4U_{\text{max}}}{H^2} z(z-H) \tag{S5}$$

Taking the derivative of the $u(x,z)$ profile at $z = H$:

$$\left.\frac{\partial u}{\partial z}\right|_{\text{H}} = \frac{12k_{\text{w}}\Delta T}{\rho_{\text{w}} L_{\text{f}} H^2 w_{\text{c}}} x \tag{S6}$$

Performing a double integral in the x and y directions, where $x = l_i$ and $y = w_{\text{r}}$:

$$F_{\text{chan}} = \frac{6\mu k_{\text{w}} w_{\text{r}} \Delta T l_i^2}{\rho_{\text{w}} L_{\text{f}} h_{\text{e}} H^2} \tag{S7}$$

The active channel length is the distance from the beginning of the channel to the edge of the ice disk. We simplify this by taking the center of the channel as an average l_i and finding the intercept point along the circle. The slope of the linear equation is found by taking $1/\tan$ of the respective wedge angle:

$$y = mx + b = \sqrt{R^2 - x^2} \tag{1}$$

where m is either 1 or 2.414 for wedge angles of 45° or 22.5°, respectively. The y-intercept is found by using the spacing of the center-to-center channels along the y-axis and iterating through for each of the channels possibly under the ice disk. Solving for x in the previous equation then dividing by $\cos(1-\alpha)$ results in l_i. A tabulated output of l_i for a half-space beneath an ice disk can be seen in Table S1.

To find the velocity of the ice disk, the viscous resisting force along the ridges is equal to the inertial force:

$$\mu_{\text{w}} \frac{\partial u}{\partial z} A_{\text{resist}} = \frac{6\mu k_{\text{w}} w_{\text{r}} \Delta T l_i^2}{\rho_{\text{w}} L_{\text{f}} h_{\text{e}} H^2} \tag{S8}$$

Table S1: Lengths of active channels underneath a half-space of the ice disk for $R_{\mathrm{ice}} = 26\,\mathrm{mm}$ for the herringbone geometry detailed in Figure 2 of the main manuscript.

Channel #	$l_{i,45^\circ}$ (mm)	$l_{i,22.5^\circ}$ (mm)
1	15.9	16.3
2	25.6	25.8
3	32.0	32.1
4	36.8	36.8
5	36.5	40.6
6	36.0	43.6
7	35.1	46.1
8	33.9	48.0
9	32.6	43.7
10	31.0	39.2
11	29.3	34.5
12	31.0	29.7
13	25.3	24.6
14	23.0	19.4
15	20.6	14.0
16	17.9	8.4
17	15.1	2.7
18	12.1	-
19	8.8	-
20	5.3	-
21	1.4	-

Assuming a linear velocity profile and no-slip on the ridge top culminates in the ratchet speed of the ice disk:

$$U_{\mathrm{ice}} = \frac{6k_{\mathrm{w}}\Delta T\bar{l}_i}{\rho_{\mathrm{w}}L_{\mathrm{f}}H^2}h^*. \tag{S9}$$

Complete derivation of model for Laplace ice slingshot on a SHPB herringbone

For the SPHB experiments, the same assumptions apply except that the filling time is now extended to an onset time to create a leading puddle long enough to create a mismatch in Laplace pressure:

$$t_{\text{onset}} = \frac{6\mu w_{\text{r}}}{h_{\text{e}}^{3}\rho_{\text{i}} g h_{\text{i}} l_{i}} \left(\frac{V_1}{n_1} + \frac{V_2}{n_2} + \frac{V_3}{n_3}\right) \tag{S10}$$

Where V_2 is the circumference around the ice disk and can be solved as a hollow cylinder:

$$V_2 \approx \pi \left(2R_{\text{ice}} l_{\text{c}} + l_{\text{c}}^{2}\right) H_{\text{i}} \tag{S11}$$

V_3 is the volume of the puddle in front simplified to a ovalish puddle from $[\pi/6, 5\pi/6]$:

$$V_3 = (3/2)\pi l_{\text{c}}^{2} R_{\text{ice}} \tag{S12}$$

Comparing the two Laplace pressures acting on the trailing and leading edge of the ice disk:

$$\Delta P = P_1 - P_2 = \gamma \left[\left(\frac{1}{l_{\text{c}}} + \frac{1}{R_{\text{ice}}}\right) - \left(\frac{1}{\infty} + \frac{1}{R_{\text{ice}}}\right)\right] = \frac{\gamma}{l_{\text{c}}} \tag{S13}$$

The contact area of the Laplace force is the height of the puddle multiplied by the puddle area in front of the ice disk between $[\pi/6, 5\pi/6]$:

$$F_{\text{cap}} \approx (2l_{\text{c}}) \left(\frac{2\pi R_{\text{ice}}}{3}\right) \frac{\gamma}{l_{\text{c}}} \approx \frac{4\pi}{3}\gamma R_{\text{ice}} \tag{S14}$$

The capillary force compared to the ice disk's inertia is simply the force divided by the ice mass:

$$\frac{4\pi}{3}\gamma R_{\text{ice}} \approx ma \tag{S15}$$

Solving directly for the acceleration:

$$a_{\mathrm{disk}} = f \frac{4\gamma}{3\rho_{\mathrm{i}} R_{\mathrm{ice}} h_{\mathrm{disk}}} \tag{S16}$$

where f is a fitting factor of 0.15.

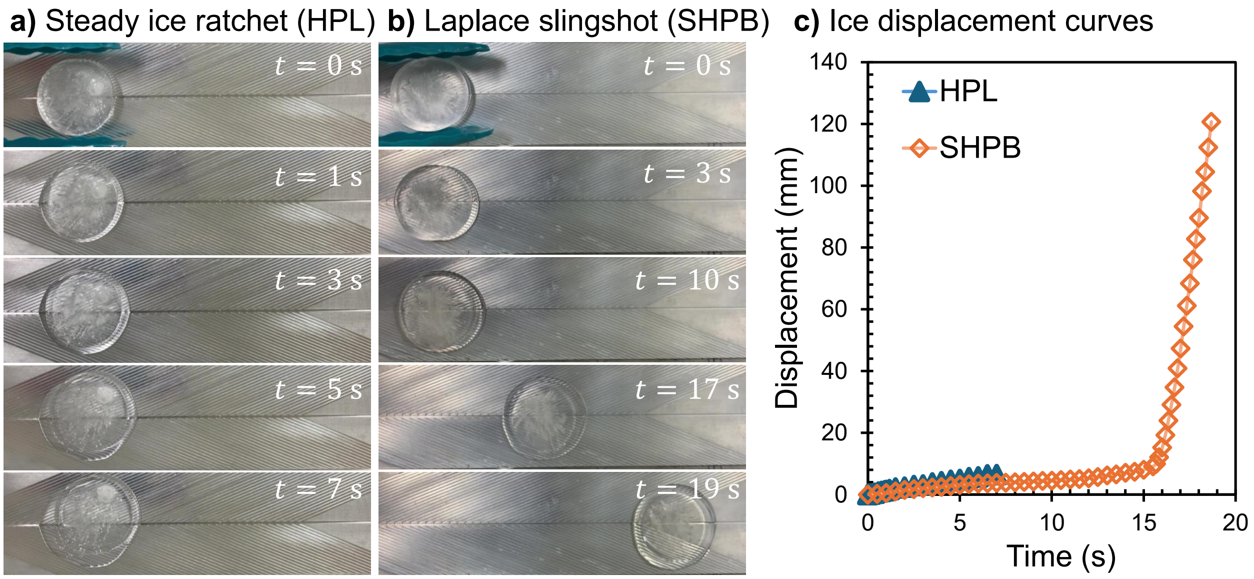

Figure S1: Representative trial of Case 1, with surface temperature $T_s = 65\,°C$, herringbone angle $\alpha = 22.5$, and channel depth $h_c = 0.25\,mm$. (a,b) Time-lapse photography of a trial on (a) an uncoated HPL herringbone, or (b) SHPB herringbone. (c) Graph of displacement versus time with the corresponding HPL trial in blue triangles and the SHPB trial in hollow orange diamonds.

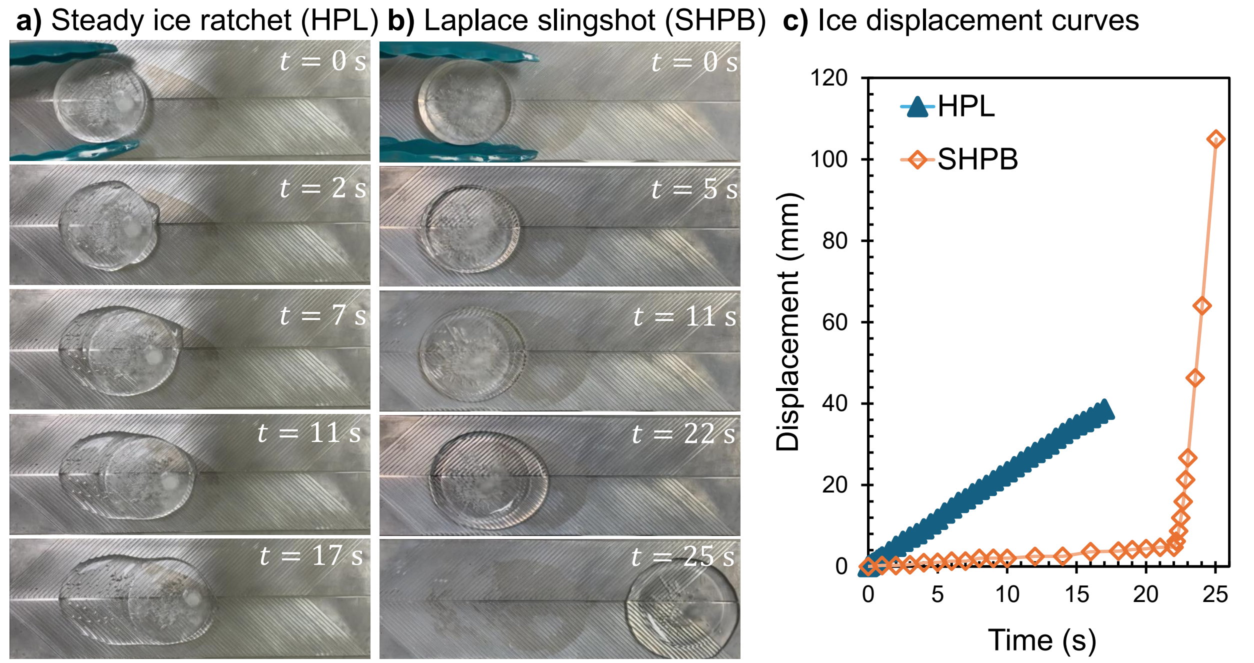

Figure S2: Representative trial of Case 2, with surface temperature $T_s = 65\,°C$, herringbone angle $\alpha = 45$, and channel depth $h_c = 0.25\,mm$. (a,b) Time-lapse photography of a trial on (a) an uncoated HPL herringbone, or (b) SHPB herringbone. (c) Graph of displacement versus time with the corresponding HPL trial in blue triangles and the SHPB trial in hollow orange diamonds.

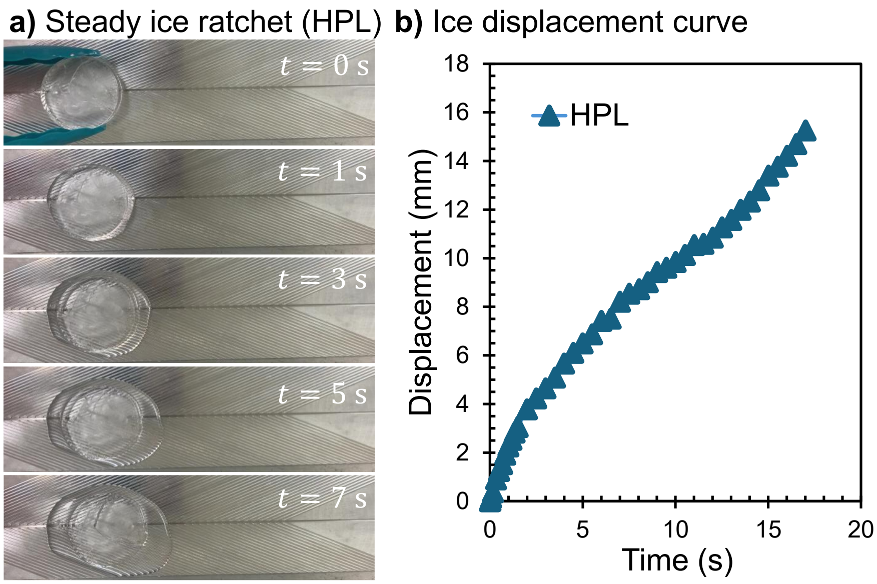

Figure S3: Representative trial of Case 3, with surface temperature $T_s = 65\,°C$, herringbone angle $\alpha = 22.5$, and channel depth $h_c = 0.5\,mm$. (a) Time-lapse photography where ice propulsion was only successful for the HPL herringbone, not the SHPB one. (b) Graph of displacement versus time for the HPL ratchet.

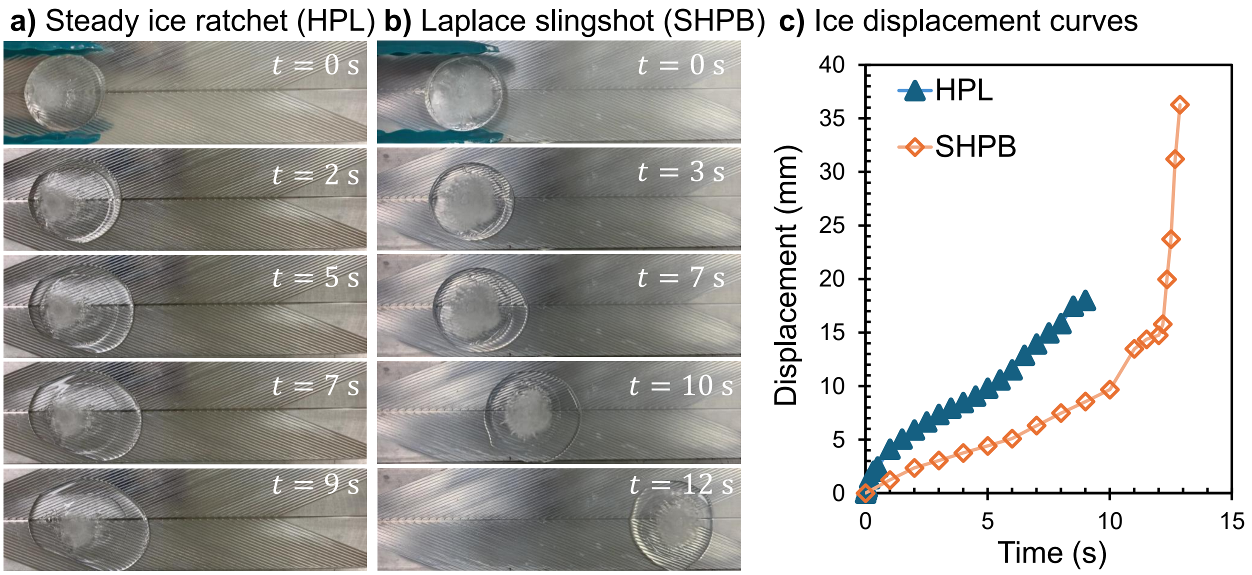

Figure S4: Representative trial of Case 4, with surface temperature $T_s = 150\,°C$, herringbone angle $\alpha = 22.5$, and channel depth $h_c = 0.25\,mm$. (a,b) Time-lapse photography of a trial on (a) an uncoated HPL herringbone, or (b) SHPB herringbone. (c) Graph of displacement versus time with the corresponding HPL trial in blue triangles and the SHPB trial in hollow orange diamonds.

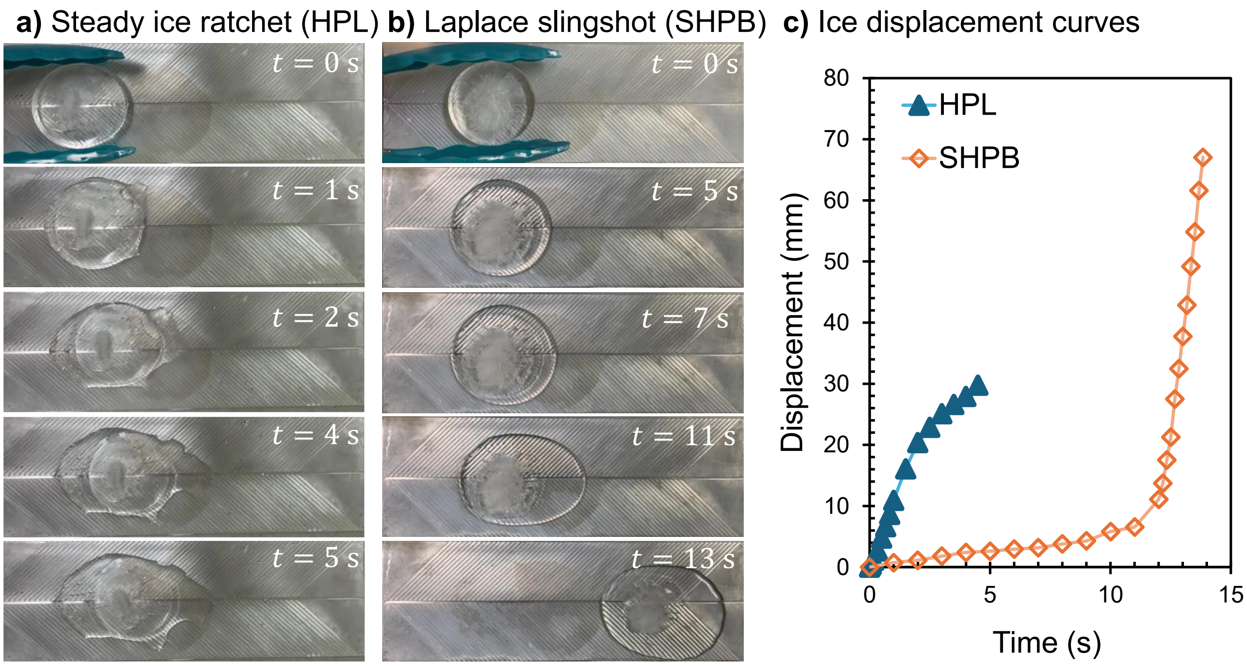

Figure S5: Representative trial of Case 5, with surface temperature $T_s = 150\,°C$, herringbone angle $\alpha = 45$, and channel depth $h_c = 0.25\,mm$. (a,b) Time-lapse photography of a trial on (a) an uncoated HPL herringbone, or (b) SHPB herringbone. (c) Graph of displacement versus time with the corresponding HPL trial in blue triangles and the SHPB trial in hollow orange diamonds.

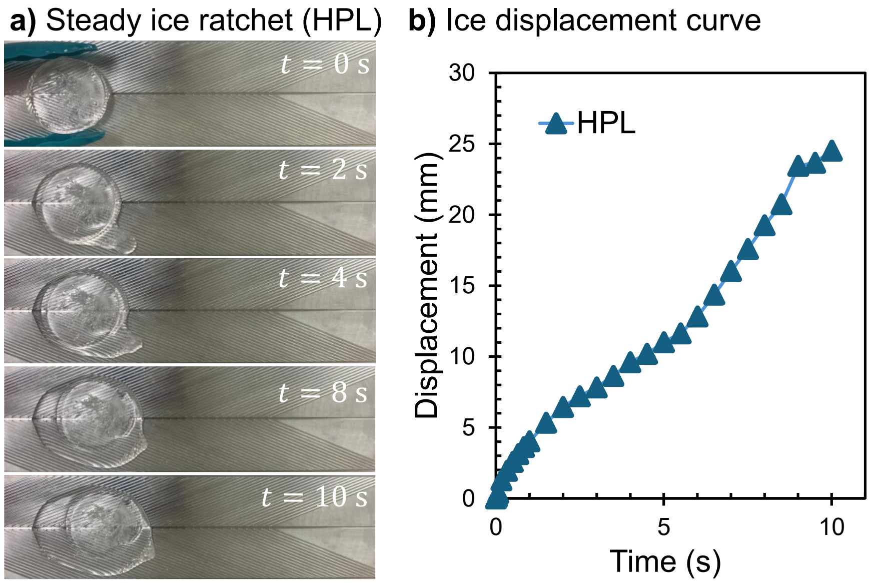

Figure S6: Representative trial of Case 6, with surface temperature $T_s = 150\,°C$, herringbone angle $\alpha = 22.5$, and channel depth $h_c = 0.5\,mm$. (a) Time-lapse photography where ice propulsion was only successful for the HPL herringbone, not the SHPB one. (b) Graph of displacement versus time for the HPL ratchet.

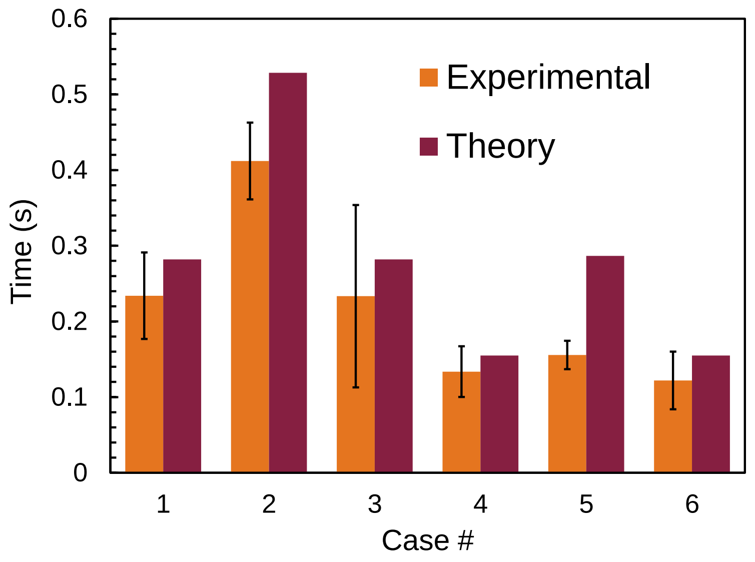

Figure S7: Side by side comparison of the experimental onset time (orange) and theoretical filling time (burgundy, Eq. 2 from main manuscript) for the initiation of ice ratcheting on the HPL herringbone. The error bars refer to the standard deviation of the average trials.

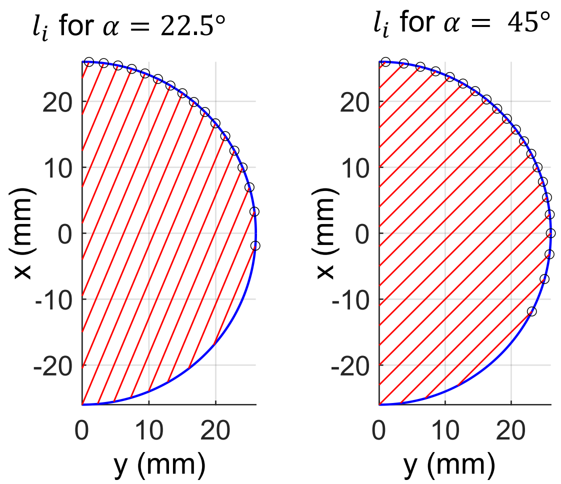

Figure S8: Graphical image of the active channel length l_i. The red lines represent the centerline of each respective channel where the total length is l_i. The x and y axes are the represented directions shown in Fig. 2 and (0,0) is the center of the ice disk. The characteristic channel length $\bar{l}_i$ for $\alpha = 22.5\,^\circ$ is 29.7 mm and for $\alpha = 45\,^\circ$ is 23.9 mm

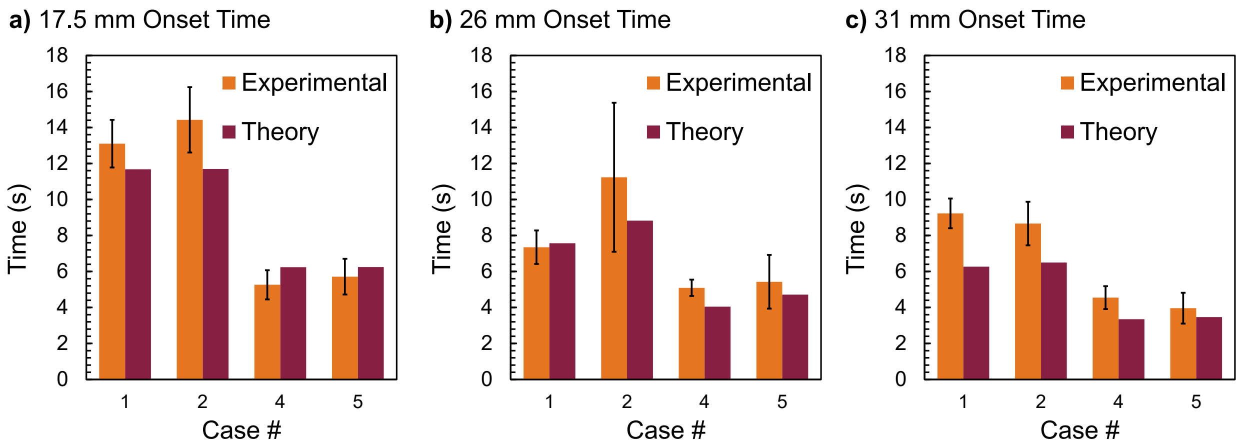

Figure S9: Side by side comparison of the experimental onset time (orange) and theoretical filling time (burgundy, Eq. 2 from main manuscript) for the initiation of ice slingshotting on the SHPB herringbone. The labels above each graph refer to the initial radius of the deposited ice disk and error bars refer to the standard deviation of the average trials.

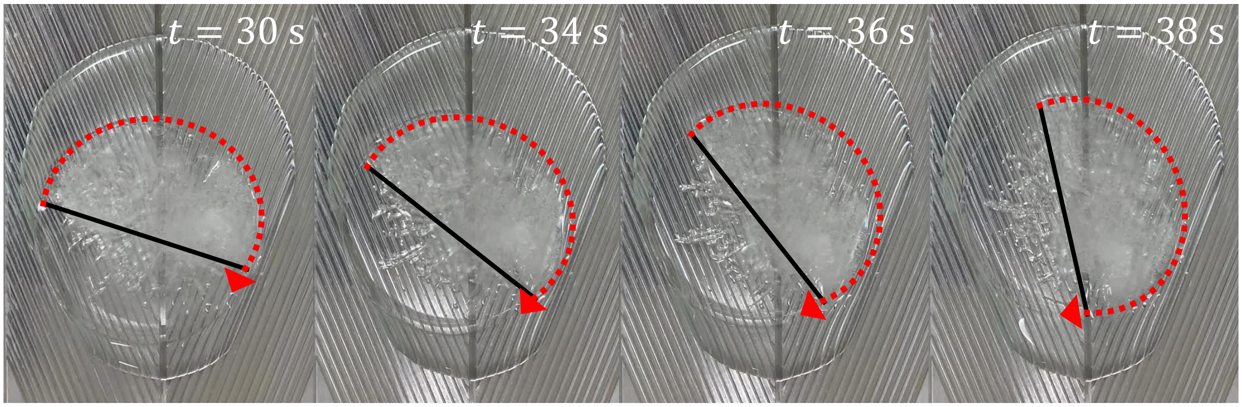

Figure S10: Top-view imaging of clockwise rotation of an ice disk on HPL herringbone with surface temperature, $T_s = 65\,°$. The ice disk rotates at the end of the ratcheting lifetime due to the entraining force becoming negligible.

Movie M1: Top-view videos of the six successful cases of ice ratcheting on the HPL herringbones. The six video sequences correspond to Cases 1–6 shown in Figures S1–S6 above. After a brief filling time ($< 1\,s$), the ice disk in question begins to ratchet along the herringbone. All videos are in real time.

Movie M2: Top-view videos of the four successful cases (Cases 1, 2, 4, and 5) of ice slingshotting on the SHPB herringbones. The four video sequences correspond to Figures S1, S2, S4, and S5 above. After the onset time to form a puddle preferentially at the front of the ice disk, Laplace tugging dislodges the partially adhered ice from the SHPB ridge tops and slingshots it across the herringbone. All videos are in real time.

Movie M3: Top-view videos for the control experiments on uniform HPL or SHPB plates without herringbones. The HPL aluminum has random movements that depends on the meltwater expansion. The SHPB has no movement at all due to the total adhesion of the ice to the surface. All videos are in real time.
